# DNA Methylomes and Epigenetic Age Acceleration Associations with Poor Metabolic Control in T1D

**DOI:** 10.3390/biomedicines9010013

**Published:** 2020-12-24

**Authors:** Raúl F Pérez, Juan Luis Fernandez-Morera, Judit Romano-Garcia, Edelmiro Menendez-Torre, Elias Delgado-Alvarez, Mario F Fraga, Agustin F Fernandez

**Affiliations:** 1 Nanomaterials and Nanotechnology Research Center (CINN-CSIC), Health Research Institute of Asturias (ISPA), Institute of Oncology of Asturias (IUOPA), and Centro de Investigación Biomédica en Red de Enfermedades Raras (CIBERER), 33011 Oviedo, Asturias, Spain; rauldiul@gmail.com; 2 Endocrinology and Nutrition Department, Hospital Vital Alvarez Buylla (HVAB), 33611 Mieres, Asturias, Spain; 3 Servicio de Anestesiologia y Reanimacion, Hospital Universitario Central de Asturias (HUCA), 33011 Oviedo, Asturias, Spain; jromano@alumni.unav.es; 4 Endocrinology and Nutrition Department, Hospital Universitario Central de Asturias (HUCA), 33011 Oviedo, Asturias, Spain; edelangot@gmail.com (E.M.-T.); eliasdelga@gmail.com (E.D.-A.); 5 Endocrinology, Nutrition, Diabetes and Obesity Unit, Instituto de Investigación Sanitaria del Principado de Asturias (ISPA), and Centro de Investigación Biomédica en Red de Enfermedades Raras (CIBERER), 33011 Oviedo, Asturias, Spain; 6 Medicine Department, Universidad de Oviedo, 33011 Oviedo, Asturias, Spain; 7 Department of Organisms and Systems Biology (B.O.S), University of Oviedo, 33011 Oviedo, Asturias, Spain

**Keywords:** type 1 diabetes, DNA methylation, PhenoAge, epigenetic aging, age acceleration, metabolic control

## Abstract

Type 1 diabetes (T1D) is an autoimmune disease that leads to insulin deficiency and hyperglycemia. Little is known about how this metabolic dysfunction, which substantially alters the internal environment, forces cells to adapt through epigenetic mechanisms. Consequently, the purpose of this work was to study what changes occur in the epigenome of T1D patients after the onset of disease and in the context of poor metabolic control. We performed a genome-wide analysis of DNA methylation patterns in blood samples from 18 T1D patients with varying levels of metabolic control. We identified T1D-associated DNA methylation differences on more than 100 genes when compared with healthy controls. Interestingly, only T1D patients displaying poor glycemic control showed epigenetic age acceleration compared to healthy controls. The epigenetic alterations identified in this work make a valuable contribution to improving our understanding of T1D and to ensuring the appropriate management of the disease in relation to maintaining healthy aging.

## 1. Introduction

Type 1 diabetes (T1D) is a widespread disease involving the autoimmune-associated destruction of β-islets in the pancreas as well as insulin deficiency. This lack of insulin leads to difficulties in glucose management, and consequently, hyperglycemia, which is a fundamental indicator for the diagnosis and development of the disease [1,2].

Several studies in the context of T1D have established that the initial glycemic environment defines a “metabolic memory”, the effects of which can persist overtime, even after a return to normal glycemic values [3,4]. In fact, T1D has become an important health concern in the elderly [5]. Although still under investigation, oxidative stress, non-enzymatic glycation of proteins, epigenetic changes, and chronic inflammation have all been proposed to play a role in terms of long-term effects on the metabolic memory [6,7].

Epigenetic marks such as DNA methylation have been shown to play major roles in the interplay between external stimuli and genetic response [8], and thus their study is of interest both for the definition of potential disease biomarkers and to shed light on the molecular mechanisms of T1D [9]. In this sense, DNA methylation has been studied in T1D on a genome-wide scale, especially in combination with genetic markers for T1D etiology [10,11,12], but little is known about the epigenetic response in T1D after disease and poor metabolic control come into play, or how these changes could be related to T1D complications [13].

In addition, the recent development of epigenetic clocks based on DNA methylation biomarkers has enabled estimates of biological age to be made that can be used to study the impact of diseases on human health [14,15]. In the context of T1D, a recent study has analyzed the association between epigenetic age, as measured by four different estimators, and the development of diabetic complications in a cohort of 499 patients [16]. Although the estimators analyzed were not completely accurate in detecting subjects with T1D who are at higher risk of developing complications, some of them displayed statistically significant associations between DNA methylation (DNAm) age and decreased kidney function, diabetic peripheral neuropathy and cardiovascular autonomic neuropathy [16].

Here, we profiled the genome-wide DNA methylation levels in whole blood from 18 adult individuals with T1D using the Illumina MethylationEPICBeadchip platform. By comparing the results with various cohorts of healthy individuals, we have been able to define robust T1D-associated DNA methylation differences in more than 100 genes, and, furthermore, we found that T1D patients showing poor metabolic control, based on the measurement of HbA1c levels, manifest statistically significant epigenetic age acceleration.

## 2. Materials and Methods

### 2.1. Human Subjects

This study had approval from the human research ethics committee of the Hospital Universitario Central de Asturias and all subjects gave written informed consent. Blood samples were extracted from patients attending Alvarez-Buylla Hospital between February and April 2018. The eighteen patients included in the study (Table 1) had all been formally diagnosed at least 15 years previously (according to ADA criteria and positive pancreatic autoimmunity) and were only receiving insulin for treatment (0.5–0.6 UI/kg/day doses). Participants were divided in two groups depending on their metabolic control level according to the Standards of Medical Care in Diabetes (Diabetes Care 2020; 43 (Suppl. 1): S66–S76). Six patients had good metabolic control, with a mean HbA1C of 6.1%, sd = 0.45, while the other twelve had shown poor metabolic control of the disease over a number of years, with mean HbA1C being 8.7%, sd = 0.62 (*p*-value = 0.0009, two-sided Wilcoxon rank-sum test). With few exceptions, six-monthly HbA1c measurements had been performed for at least 15 years for all patients. All samples were assayed blind. In a later evaluation we also classified poor metabolic control patients into smokers (active or recently having stopped smoking) and non-smokers because of the serious clinicopathological implications of tobacco smoking (at least 10 packs/year) (Table 1 and Supplementary Table S1). To validate the results by bisulfite pyrosequencing, an additional cohort of 8 controls and 10 patients was collected from the Hospital Universitario Central de Asturias from 1 to 18 September 2020. Inclusion criteria for patients were the same as for the first cohort.

### 2.2. External Databases

Illumina Infinium MethylationEPIC external datasets for whole blood of adult individuals were retrieved from 2 different sources: GSE123914 [17,18], which corresponds to 35 healthy female individuals (mean age 58.5 yr, sd = 3.5 yr) which were used in a study measuring DNA methylation variability over a 1-year period. Two measurements were available for each sample, but only those corresponding to the year 2013 were retrieved (*n* = 35); and GSE131433 [19], which corresponds to 440 individuals from a cohort of newborn and adult individuals conceived by assisted reproductive technologies (ART). From this cohort, the control mixed-sex non-ART adults were selected (*n* = 75), but 1 of the individuals was discarded due to discordance between specified and methylation-predicted sex (GSM3780521_202817030128_R08C01), leading to a total of 74 individuals (51 female, 23 male, aged 18 to 35 yr).

### 2.3. DNA Extraction and Illumina Infinium MethylationEPICBeadChip

DNA was extracted from whole blood by a standard phenol-chloroform protocol. Bisulfite conversion was performed using the EZ-96 DNA Bisulfite Zymo Research conversion protocol. The Illumina Infinium HD Methylation Assay protocol was followed by hybridizing samples to Illumina Infinium Methylation EPIC BeadChips.

### 2.4. Methylation Data Preprocessing and Analyses

All preprocessing was carried out through the statistical software R (v3.5.2, Vienna, Austria). MethylationEPIC data were imported using the *minfi* package (v1.28.3) [20] and background-corrected with the NOOB method [21]. Extracted β-values were normalized by the BMIQ method [22]. Quality control and correction of genetic variation was performed by filtering the following probes: those with detection *p*-value > 0.01 in any sample, sex chromosome probes, those with SNPs with MAF ≥ 0.01 at their CpG site or their single-base extension (SBE) site (dbSNP v147), and cross-reactive and multimapping probes [23]. A total of 766127 probes were common to the 3 datasets and were those that were finally analyzed.

Differentially methylated probes (DMPs) were calculated using the *limma* package (v3.38.3) [24]. Prior to the model fitting, β-values were transformed to M-values, and quantile-normalization (*normalizeQuantiles*function) was performed to minimize global-level population differences between the cohorts. Then, linear models were used by fitting methylation levels as the dependent variable and group (T1D cohort or external cohort) as the independent variable. Additionally, possible batch or confounder effects, including potential population bias, were handled by surrogate variable analysis (SVA) using the package sva (v3.30.1) and adding the first 3 surrogate variables found as covariates to the models [25]. In order to define robust, biologically relevant DMPs, we used strict criteria and only those probes with Benjamini& Hochberg adjusted *p*-value < 0.05, logFC change in M-value > 0.5 and mean difference in β-values > 0.2 were selected. Furthermore, the two comparisons (T1D vs GSE123914 and T1D vs GSE131433) were made in parallel, and only the common DMPs found to be significant in both analyses were selected. Finally, probes were annotated to genes and genomic locations using the *IlluminaHumanMethylationEPICanno.ilm10b4.hg19 package* (v0.6.0). Exon- and exon boundary annotations were grouped within gene bodies.

DNA methylation ages were estimated by the DNA Methylation Age Calculator tool (https://dnamage.genetics.ucla.edu/home) for the pan tissue [26], GrimAge [27] and PhenoAge [14] clocks. To define epigenetic age acceleration, the predicted values were regressed on chronological age and sex by linear model fitting. The residuals thus obtained represent epigenetic age acceleration values, which take into account chronological and sex differences [28].

### 2.5. Bisulfite Pyrosequencing

Bisulfite pyrosequencing was used to measure the DNA methylation status of 5 representative CpG sites (cg08272597, cg27176729, cg00753540, cg14551152, cg12269782; see Table S3 for primer information) in an independent cohort of 18 subjects (10 T1D cases and 8 controls) attending the Hospital Universitario Central de Asturias. DNA was extracted from blood samples following a standard phenol-chloroform protocol. Bisulfite conversion of the isolated DNA was performed with the EZ DNA methylation-gold kit (Zymo research, Irvine, CA 92614, USA). After PCR amplification, pyrosequencing was performed using PyroMark Q24 reagents and a vacuum prep workstation, equipment and software (Qiagen, Hilden, Germany).

## 3. Results

### 3.1. DNA Methylation Differences Associated with T1D in Whole Blood

In order to define any DNA methylation differences associated with T1D in whole blood, we measured the genome-wide DNA methylation levels of 18 individuals with T1D (Table 1 and Table S1) using Illumina MethylationEPICBeadChip arrays.

**Figure 1:**
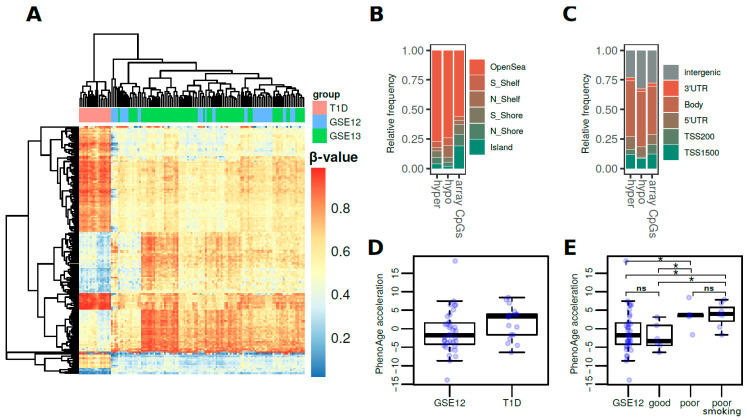
(**A**) Heatmap showing the methylation β-values for the 166 common significantly differentially-methylated CpG sites in the T1D versus control cohorts. (**B**) Barplots showing the relative distribution of DMPs at CpG island locations. (**C**) Barplots showing the relative distribution of DMPs at genetic locations. (**D**) Boxplots representing the DNAm PhenoAge values of the T1D cohort and the GSE123914 cohort. (**E**) Boxplots representing the DNAm PhenoAge acceleration for the T1D cohort by subgroup with respect to metabolic control (* = *p*-value < 0.05; ns = non-significant; for two-tailed Wilcoxon rank-sum tests).

To define robust T1D-associated DNA methylation differences, we compared our data to EPIC whole blood array data from 2 independent external cohorts of healthy adult individuals (see Methods, *n* = 35 and 74) (Table 1). To control for potential unwanted sources of variability, a surrogate variable analysis (SVA) was performed and surrogate variables were added as covariates to the models (see Methods). From the 2 comparisons we derived 166 common significantly differentially-methylated probes (DMPs) (average difference in β-values > 0.2, FDR < 0.05) (Table S2 which displayed similar T1D-associated differences and perfectly distinguished T1D patients (Figure 1A). These consisted of 74 hyper- and 92 hypomethylated CpG sites, which were predominantly found at open sea locations (Figure 1B) regardless of their direction of change (ORs = 2.7 and 2.3 for hyper- and hypomethylation, respectively, *p*-value < 0.01, two-sided Fisher’s tests). On the other hand, no substantial differences were observed regarding gene locations when compared to the array background (Figure 1C).

To further validate these results, we used bisulfite pyrosequencing to analyze 5 differentially methylated CpGs in another external independent cohort of 18 subjects (10 T1D cases and 8 controls; see Methods). We observed concordant trends of change for all of the evaluated CpGs, most of which approached, but not reached statistical significance because of the limited number of observations (Figure S1).

These DMPs were associated with more than 100 genes, and it is worth noting that these included genes that code for epigenetic enzymes such as SET And MYND Domain Containing 3 Histone methyltransferase (*SMYD3*) which is associated with oxidative stress response; methionine sulfoxide reductase B3 (*MRSA3B*), genes associated with immune response such as major histocompatibility gene *HLA-DQB1*; and genes associated with glucose metabolism such as phosphatase and tensin homolog (*PTEN*) and Glycogen synthase kinase 3 beta (GSK3B) (Table S2).

### 3.2. DNA Methylation Age Is Accelerated in T1D and Is Related to Glycemic Control

Finally, we used Horvath’s healthspan epigenetic clock (PhenoAge) to calculate the DNAm ages of the T1D cohort and the GSE123914 cohort, which included chronological age data. To determine DNAm PhenoAge acceleration, the PhenoAge values were adjusted for chronological age and sex (see Methods) [28]. When looking at the association between DNAm age and T1D pathology, we first observed a non-significant trend of increased epigenetic age acceleration for T1D patients (2.5 pheno-year increase, *p*-value = 0.054, two-sided Wilcoxon rank-sum test) (Figure 1D). Subsequently, though, we classified the T1D patients by HbA1c serum levels (Table 1 and Table S1), which is the gold standard measurement for the assessment of glycemic control. Interestingly, this led to the finding that only individuals with poor glycemic control, regardless of smoking status, showed a significant increase in epigenetic age acceleration compared with healthy controls (4.4 and 4.6 pheno-year increase, *p*-values = 0.047 and 0.014, respectively, two-sided Wilcoxon rank-sum test) (Figure 1E). In other words, there were no significant differences between T1D patients with good metabolic control and healthy controls. (*p*-value = 0.663, two-sided Wilcoxon rank-sum test) (Figure 1E). We further analyzed the pan-tissue and GrimAge clocks (see Methods) to find much smaller trends of epigenetic age acceleration (Figure S2). In particular, we observed that GrimAge acceleration was especially influenced by smoking status (Figure S2).

## 4. Discussion

In this work we profiled the DNA methylomes of 18 adult T1D patients using MethylationEPICarrays. Although genome-wide methylomic profiling has been performed in subjects with T1D diabetes, most have not focused on either the involvement of metabolic control in the appearance of epigenetic changes or its association with epigenetic age [11,17,29]. Our results reveal robust DNA methylation differences associated with T1D as compared to two independent external cohorts of healthy individuals. We further validated the results through bisulfite pyrosequencing, in an additional independent cohort of samples selected following the same inclusion criteria as for the methylation arrays, demonstrating the consistency of the changes identified in this work. These changes are linked to several genes related to epigenetic modifiers, oxidative stress and immune responses, as well as glucose metabolism, and might well underlie the long-term effects of these molecular mechanisms on metabolic memory [6,7].

Importantly, we detected an increase in epigenetic age acceleration associated with T1D in patients with poor glycemic control, based on glycated hemoglobin (HbA1c) levels. Since only patients with poor metabolic control manifested diabetic complications (microangiopathy, diabetic nephropathy, retinopathy, or neuropathy) (Table S1), their occurrence may also help explain the observation of age acceleration.

From all the methods available to measure DNAm age, we first focused on the PhenoAge because in a recent study it has been shown to be one of the best methods of analyzing T1D patients who are at higher risk of developing complications, and, in particular, was positively associated with HbA1c levels and T1D duration, which are the two clinical variables used for patient selection in our study [16]. In fact, this same work reported that only PhenoAge was positively correlated with HbA1c levels, a major risk indicator for diabetic complications [16]. In our work, we not only identified an increase in epigenetic age acceleration linked to worse HbA1c levels in T1D patients, but, and more importantly, we also observed that patients with good metabolic control do not show significant differences in age acceleration values compared to healthy controls, suggesting that metabolic control may help disease management within the context of aging. Additionally, we also studied the pan-tissue and GrimAge clocks to find much less association between their age acceleration predictions and our phenotypes. This observation suggests that some epigenetic clocks may be more appropriate than others as biomarkers for specific pathologies.

In conclusion, the epigenetic alterations identified in our work provide a list of new potential molecular biomarkers with clinical utility for T1D prognosis, which also progress our molecular understanding of the disease. Moreover, the increase in epigenetic age acceleration in T1D patients showing poor metabolic control highlights the importance of tracking and monitoring these patients to ensure the appropriate management of the disease in relation to maintaining healthy aging.

## Figures and Tables

**Table 1:** Clinical information for the control and T1D cohorts. Data are represented as means ± SD.

	*n*	Age (Years)	Male/Female	T1D Duration (Years)	Age of Onset (Years)	HbA1c % (mmol/mol)
T1D patients						
Good metabolic control	6	43.2 ± 10.9	2 / 4	21.8 ± 14.4	21.3 ± 4.4	6.1 ± 0.5 (44.3 ± 2.4)
Poor metabolic control	12	48.3 ± 7.0	4 / 8	24.8 ± 9.3	23.5 ± 8.1	8.7 ± 0.6 (68.9 ± 8.8)
Poor metabolic control + nonsmoker	5	48.0 ± 6.2	1 / 4	28.2 ± 11.5	19.8 ± 6.9	8.4 ± 0.6 (61.4 ± 5.9)
Poor metabolic control + smoker	7	48.4 ± 8.0	3 / 4	22.3 ± 7.3	26.1 ± 8.4	8.8 ± 0.7 (74.3 ± 6.1)
Controls						
GSE123914	35	58.5 ± 3.5	0/35			
GSE131433	74	18-35	23/51			

## Supplementary Materials

The following are available online at https://www.mdpi.com/2227-9059/9/1/13/s1, Table S1: Extended clinical information for the T1D cohort. Table S2: List of T1D-associated differentially-methylated probes common to the 2 control cohorts, with information related to genomic location, methylation change and associated genes. Table S3. Information regarding the primers used for the pyrosequencing validation of differentially methylated CpGs. Figure S1. (A) Heatmap showing the array β-values of the differentially methylated CpGs across T1D and control cohorts which were chosen for external validation. (B) Boxplots showing the bisulfite pyrosequencing values of the differentially methylated CpGs chosen for validation, in an external independent cohort of T1D (*n* = 10) and control (*n* = 8) subjects. *p*-values are shown for one-tailed Wilcoxon rank-sum tests. Figure S2. (A) Boxplots representing the DNAm Pan-tissue acceleration values for the T1D cohort and the GSE123914 cohort. On the right, the T1D cohort is divided into subgroups (good or poor metabolic control with or without smoking). (B) Boxplots representing the DNAm GrimAge acceleration values for the T1D cohort and the GSE123914 cohort. On the right, the T1D cohort is divided into subgroups (good or poor metabolic control with or without smoking).

## Abbreviations

DMPs	Differentially methylated probes
DNAm	DNA methylation
HbA1c	Glycated hemoglobin
SVA	Surrogate variable analysis
T1D	Type 1 diabetes
