# Supplementary material for: DNA Methylomes and Epigenetic Age Acceleration Associations with Poor Metabolic Control in T1D
Source: Biomedicines. 2020 Dec 24;9(1):13. doi: 10.3390/biomedicines9010013 (PMC7824441; doi:10.3390/biomedicines9010013)
Supplement: Supplementary file 1 [file biomedicines-09-00013-s001.zip › Supplementary Figure 2.pdf]

A

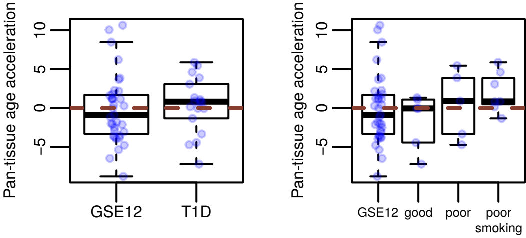

Pan-tissue age acceleration
10
5
0
−5
GSE12
T1D
Pan-tissue age acceleration
10
5
0
−5
GSE12
good
poor
poor smoking

B

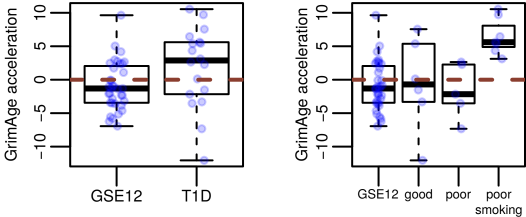

GrimAge acceleration
10
5
0
−5
−10
GSE12
T1D
GrimAge acceleration
10
5
0
−5
−10
GSE12
good
poor
poor smoking
