# Supplementary figures and images for: DNA Methylomes and Epigenetic Age Acceleration Associations with Poor Metabolic Control in T1D
Source: Biomedicines. 2020 Dec 24;9(1):13. doi: 10.3390/biomedicines9010013 (PMC7824441; doi:10.3390/biomedicines9010013)

**A**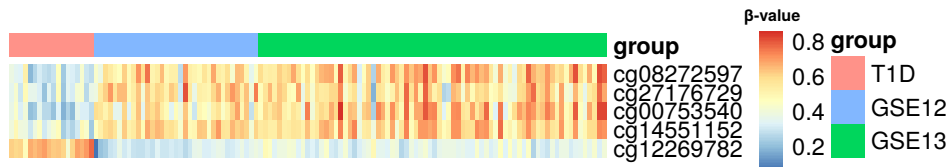**B**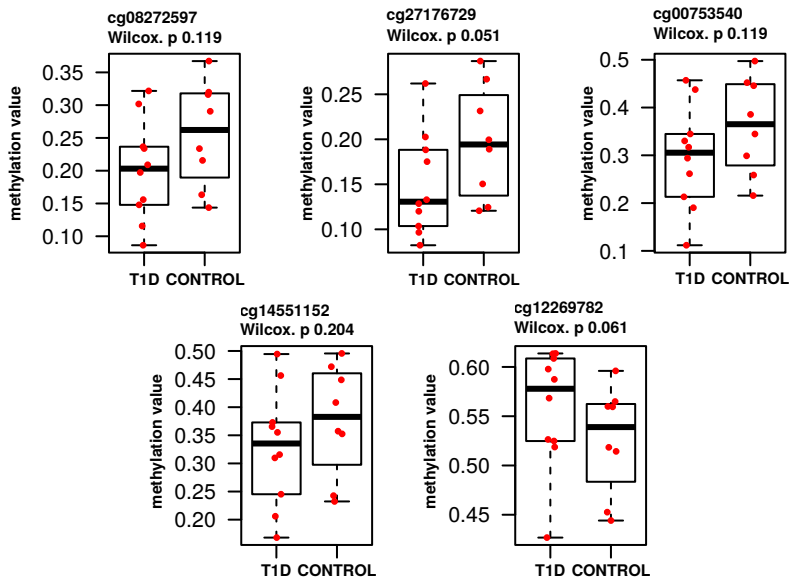

Supplement: Supplementary file 1 [file biomedicines-09-00013-s001.zip › Supplementary Figure 1.pdf]
